# Racial residential segregation shapes the relationship between early childhood lead exposure and fourth-grade standardized test scores

**DOI:** 10.1073/pnas.2117868119

**Published:** 2022-08-15

**Authors:** Mercedes A. Bravo, Dominique Zephyr, Daniel Kowal, Katherine Ensor, Marie Lynn Miranda

**Affiliations:** ^a^Global Health Institute, Duke University, Durham, NC 27710;; ^b^Children’s Environmental Health Initiative, University of Notre Dame, South Bend, IN 46556;; ^c^Department of Statistics, Rice University, Houston, TX 77005;; ^d^Department of Applied and Computational Mathematics and Statistics, University of Notre Dame, South Bend, IN 46556

**Keywords:** racial residential segregation, cognitive outcomes, lead exposure, standardized test scores

## Abstract

Racial/ethnic disparities in academic performance may result from a confluence of adverse exposures that arise from structural racism and accrue to specific subpopulations. This study investigates childhood lead exposure, racial residential segregation, and early educational outcomes. Geocoded North Carolina birth data is linked to blood lead surveillance data and fourth-grade standardized test scores (n = 25,699). We constructed a census tract-level measure of racial isolation (RI) of the non-Hispanic Black (NHB) population. We fit generalized additive models of reading and mathematics test scores regressed on individual-level blood lead level (BLL) and neighborhood RI of NHB (RINHB). Models included an interaction term between BLL and RINHB. BLL and RINHB were associated with lower reading scores; among NHB children, an interaction was observed between BLL and RINHB. Reading scores for NHB children with BLLs of 1 to 3 µg/dL were similar across the range of RINHB values. For NHB children with BLLs of 4 µg/dL, reading scores were similar to those of NHB children with BLLs of 1 to 3 µg/dL at lower RINHB values (less racial isolation/segregation). At higher RINHB levels (greater racial isolation/segregation), children with BLLs of 4 µg/dL had lower reading scores than children with BLLs of 1 to 3 µg/dL. This pattern becomes more marked at higher BLLs. Higher BLL was associated with lower mathematics test scores among NHB and non-Hispanic White (NHW) children, but there was no evidence of an interaction. In conclusion, NHB children with high BLLs residing in high RINHB neighborhoods had worse reading scores.

## Significance

Racial residential segregation (RRS) – defined here as the geographic separation of Black individuals and communities from other racial/ethnic groups into separate, unequal neighborhoods – fosters environments inimical to health through disinvestment of resources and concentration of disadvantages. Neighborhood environments influence children’s health and development, but relationships between RRS and cognitive development are poorly understood. We find that: (1) non-Hispanic Black children were more likely to experience multiple adverse exposures in early childhood, and (2) among non-Hispanic Black children, high levels of RRS augmented the detrimental effect of elevated blood levels on reading test scores. Non-linear models were used to model exposure to lead and RRS, and their interaction.

In the United States, there are longstanding racial/ethnic disparities in academic performance and educational attainment, evidenced by lower high school and college graduation rates among some racial/ethnic groups, particularly Hispanic and non-Hispanic Black (NHB) individuals (1). These disparities, sometimes referred to as the “achievement gap,” may emerge in early childhood and persist over time (1). Academic performance in early childhood predicts later-life educational outcomes, including high school graduation (1), which relates to measures of health, socioeconomic status, and well-being in adulthood (2). Disparities in academic achievement in elementary school children may be preventable and warrant attention as potentially important foci for early intervention.

It is widely agreed that positive and negative outcomes are determined by multiple forces, acting across the life course, yet surprisingly little is known about the interactions of those forces. A plethora of evidence demonstrates that lead exposure in young children, even at low levels, is associated with learning deficits and lower scores on intelligence and standardized tests (3–7). Adverse effects of childhood lead exposure persist into adulthood, affecting intelligence and socioeconomic status (SES) (8). Environmental exposures such as lead may be elevated in communities experiencing a multitude of disadvantages (9) such as poverty, deprivation, or segregation (10).

Neighborhood characteristics, and poverty specifically, have been shown to relate to cognitive development in children (11), including verbal ability (12). However, there is a dearth of work examining relationships between cognitive and developmental outcomes and neighborhood characteristics beyond measures of SES. In particular, racial residential segregation (RRS)—the geographic separation of Black individuals and communities from other racial/ethnic groups (13)—has, through the concentration of poverty and poor physical and social environments, resulted in distinctive environments that may underlie racial disparities in health outcomes (14). RRS is linked with adverse health outcomes, including infant and adult mortality (15–19), adverse pregnancy outcomes (e.g., preterm birth, low birth weight) (20–22), and chronic diseases such as hypertension and type 2 diabetes (23–26). These associations often persist even after controlling for SES, suggesting that SES may not fully capture all pathways and processes through which segregation affects neighborhood environments, resources, and residents. Yet few, if any, studies have explored associations between neighborhood RRS and cognitive outcomes in children.

Despite the potential for adverse neighborhood conditions to amplify health, cognitive, and developmental effects of lead (or other environmental exposures), effects of lead exposure are seldom evaluated alongside neighborhood contextual factors (e.g., deprivation, RRS). Thus, relationships and interactions among lead exposure and neighborhood context, and the impact on cognitive and developmental outcomes, may be important but are not thoroughly understood. Murine models of stress and lead exposure indicate that depauperate environments exacerbate the neurological impact of lead exposure (27). Similarly, human epidemiologic models suggest that stress exacerbates the impact of air pollution on asthma severity (28). The mechanism(s) for these combined effects are not fully understood, with potential explanations being priming of the stress response pathway, priming the inflammatory pathway, or both.

Here, we examine the combined effect of social (RRS) and environmental (lead) exposures on key developmental outcomes. To do so, we construct a longitudinally linked spatiotemporal dataset that tracks children from time of birth to time of fourth-grade end-of-grade testing by linking multiple, statewide administrative datasets. We then estimate associations between key exposures and end-of-grade standardized test scores in reading and mathematics for fourth-grade public school students in North Carolina, including interaction terms between individual-level lead exposure and a neighborhood-level RRS measure (racial isolation of NHB individuals [RI_NHB_]) to evaluate whether one exposure potentially augments susceptibility to another.

## Results

### Descriptive Statistics.

The median birthweight percentile for gestational age was 36.7 for NHB children compared with 53.0 for non-Hispanic White (NHW) children (Table 1). The median blood lead level (BLL) among NHB children (4.0 μg/dL) was higher than that for NHW children (3.0 μg/dL). A higher percentage of NHB children (76.4%) resided in urban areas compared to NHW children (70.7%). NHB mothers were less likely than NHW mothers to report smoking during pregnancy (11.5% vs. 21.8%) and were younger at the time of their child’s birth (median age, 23.0 vs. 26.0 y). NHB mothers were more likely to not have graduated from high school compared to NHW mothers (26.2% vs. 18.2%) and were more likely to be unmarried (72.3% vs. 25.1%). Among NHB children, 82.9% experienced economic disadvantage (i.e., participated in the free and reduced-price lunch program) compared to 44.1% of NHW children. At time of birth and standardized testing, NHB children resided in census tracts with lower median household incomes compared to NHW children. On average, at birth and standardized testing, NHB children resided in census tracts with higher levels of RI_NHB_ compared to NHW children. The RI_NHB_ distributions for NHB versus NHW children have limited overlap: for example, the 75th percentile (i.e., top of the interquartile range) of RI_NHB_ at time of standardized testing for NHW children (0.23) was equal to the 25th percentile of RI_NHB_ for NHB children (Table 1). The correlation between RI_NHB_ at birth and RI_NHB_ at time of standardized testing was 0.70 and 0.53 among NHB children and NHW children, respectively.

**Table 1.** Summary statistics of North Carolina fourth-grade students linked to birth certificate data and blood lead screening records, by race/ethnicity

Variable	All children (*n* = 25,699)	NH Black children (*n* = 9,909)	NH White children (*n* = 15,790)	*P* value
Reading test score, mean (SD)	346.8 (8.65)	343.0 (7.87)	349.2 (8.26)	<0.001
Mathematics test score, mean (SD)	352.0 (8.37)	348.2 (7.60)	354.3 (7.97)	<0.001
Child characteristics				
Birthweight percentile for gestational age, median (IQR)	45.7 (22.4–71.9)	36.7 (16.8–61.2)	53.0 (28.3–76.6)	<0.001
Blood lead test result (µg/dL), median (IQR)	3.0 (2.0–5.0)	4.0 (3.0–5.0)	3.0 (2.0–4.0)	<0.001
Children residing in urban census tracts (at time of standardized test), *n* (%)	18,733 (72.9)	7,569 (76.4)	11,164 (70.7)	<0.001
Male sex, *n* (%)	12,748 (49.6)	4,835 (48.8)	7,913 (50.1)	0.041
Computer use, *n* (%)				<0.001
None	9,100 (35.4)	3,783 (38.2)	5,317 (33.7)	
Some	14,885 (57.9)	5,174 (52.2)	9,711 (61.5)	
Always	1,714 (6.7)	952 (9.6)	762 (4.8)	
Economic disadvantage, *n* (%)*	15,172 (59.0)	8,213 (82.9)	6,959 (44.1)	<0.001
Year of end-of-grade standardized test, *n* (%)				0.135
2010	17,072 (66.4)	6,527 (65.9)	10,545 (66.8)	
2011	8,627 (33.6)	3,382 (34.1)	5,245 (33.2)	
Maternal characteristics				
Reported smoking during pregnancy, *n* (%)	4,580 (17.8)	1,135 (11.5)	3,445 (21.8)	<0.001
Age at time of child’s birth (y), median (IQR)	25.0 (21.0–30.0)	23.0 (20.0–28.0)	26.0 (22.0–31.0)	<0.001
Educational attainment at child’s birth, *n* (%)				<0.001
No high school diploma	5,474 (21.3)	2,600 (26.2)	2,874 (18.2)	
High school diploma	15,756 (61.3)	6,435 (64.9)	9,321 (59.0)	
College diploma	4,469 (17.4)	874 (8.8)	3,595 (22.8)	
Unmarried at time of birth	11,131 (43.3)	7,167 (72.3)	3,964 (25.1)	<0.001
Neighborhood characteristics (census tract)				
RI, median (IQR)				
Time of birth	0.21 (0.11–0.34)	0.34 (0.22–0.49)	0.14 (0.074–0.25)	<0.001
Time of end-of-grade test	0.20 (0.093–0.35)	0.35 (0.23–0.49)	0.13 (0.065–0.23)	<0.001
Median household income ($) in the last 12 mo, median (IQR)				
Time of birth	36,136 (28,894–43,929)	30,625 (22,500–38,226)	38,750 (32,563–46,633)	<0.001
Time of test	43,262 (32,433- 55,375)	36,711 (26,196–48,125)	46,713 (36,840–59,268)	<0.001

Summary statistics are shown for North Carolina births in 2000 linked to end-of-grade standardized testing records from two school years: 2010/2011 and 2011/2012. Cell counts and percentages are presented except in the case of continuous variables, where the mean (SD) or median (interquartile range [IQR]) are given as indicated next to the variable name, for normally distributed and nonnormally distributed variables, respectively. Maternal variables are based on reported maternal characteristics at time of the child’s birth. The χ^2^ test was used to test for differences by race group for categorical variables. T tests were used for continuous standardized test scores which were approximately normally distributed. For other continuous variables, the Wilcoxon rank sum test was used to test for differences by race group. Urbanicity was determined based on Rural-Urban Commuting Area (RUCA) codes (42).

^*^A child was considered to have economic disadvantage if they participated in the free/reduced-price school lunch program in fourth-grade, at time of end-of-grade testing.

### Reading Scores.

Due to high correlation between RI_NHB_ at birth and RI_NHB_ at standardized testing, we fit separate models to adjust for each of these variables; that is, one model that adjusts for neighborhood RI_NHB_ at birth and another that adjusts for neighborhood RI_NHB_ at standardized testing. Results from race-stratified generalized additive models for reading test scores that adjust for RI_NHB_ at time of standardized testing are presented in the main text; corresponding results for models that adjust for RI_NHB_ at time of birth are presented in *SI Appendix*. Results from adjusted models with an interaction term (BLL × RI_NHB_) are presented if the interaction was significant (*P* < 0.05); otherwise, results from the adjusted model without an interaction term are presented. We report adjusted and interaction model results for reading scores for NHW and NHB children, respectively.

**Fig. 1.**
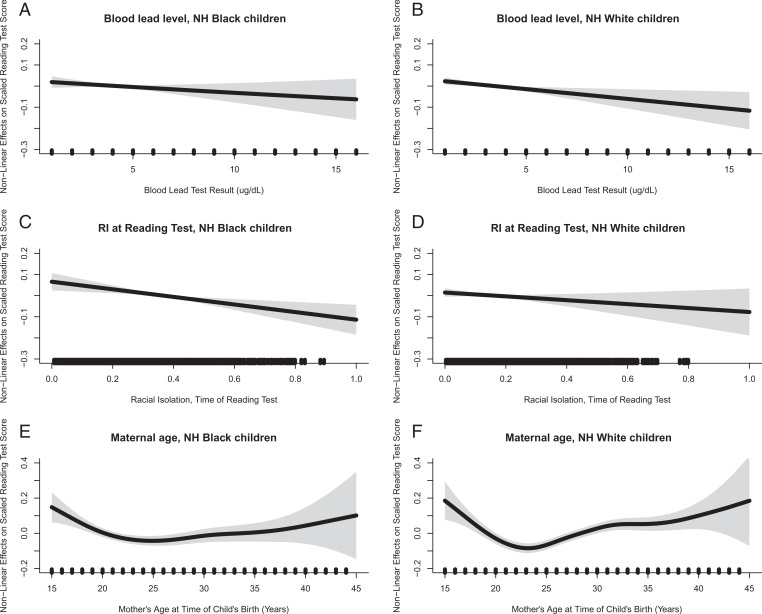
Nonlinear effects and predicted scaled end-of-grade standardized test scores in reading. The association between blood lead level and scaled reading test scores is shown for NHB children (*A*) and NHW children (*B*); the association between neighborhood racial isolation at time of standardized testing and scaled reading test scores is shown for NHB children (*C*) and NHW children (*D*); the association between maternal age at time of child’s birth and scaled reading test scores is shown for NHB children (*E*) and NHW children (*F*).

For smoothed (nonlinear) variables, we report on the complexity of the smoother (effective degrees of freedom) and the statistical significance of the smooth term (*P* value, F-statistic) in Table 2. The *P* values correspond to the hypothesis that the nonlinear function is zero everywhere, so rejection of the null hypothesis indicates an association between the nonlinear effects and standardized test scores for at least some values of that variable. The relationship between each of the smooth terms and scaled reading test scores is provided in Fig. 1. These plots depict the possibly nonlinear relationship between the respective test score and the nonlinear variables and include pointwise 95% CIs.

**Table 2.** Results of the generalized additive model regression for end-of-grade test scores in reading: nonlinear variables

Nonlinear variable	NHB children			NHW children		
	edf	*P* value	F-statistic	edf	*P* value	F-statistic
BLL	1.057	0.179	1.882	1.004	0.008	6.966
RI at time of end-of-grade test	1.002	0.001	10.478	1.021	0.147	2.044
Maternal age	3.575	0.003	3.904	5.285	<0.001	9.132
BLL × RI at time of end-of-grade test	5.472	0.003	3.007	—	—	—

Effective degrees of freedom (edf), F-statistic, and *P* value are reported instead of coefficients for nonlinear effects. edf represents the complexity of the smooth: an edf of 1 is equivalent to a straight line, an edf of 2 is equivalent to a quadratic curve, and so on, such that higher edf values describe more “wiggly” curves. Furthermore, an edf < k − 1 indicates that k is sufficiently large. The F-statistic is a test statistic used in an analysis of variance to test overall significance, which produces the *P* value. The table values are approximate, thus it is important to visualize the model to check the identified relationships (50). Dashes (—) indicate that the model did not include an interaction term.

Briefly, BLL was associated with reading test scores among NHW children, but not NHB children (note that the main effect for BLL was significant in the adjusted model for NHB children, but not in the interaction model for NHB children presented here). RI_NHB_ was associated with reading test scores among NHB children but not NHW children. An interaction between BLL and RI_NHB_ was observed for NHB children only. Maternal age was associated with reading test scores for children of both races.

The plots of BLL (Fig. 1 *A* and *B*) were linear for both races and indicate that higher concentrations of blood lead were associated with lower test scores, although this relationship only achieved statistical significance for NHW children.

The plots of neighborhood RI_NHB_ at time of standardized testing (Fig. 1, *C* and *D*) were relatively linear for both NHB and NHW and indicate that higher levels of neighborhood RI_NHB_ may be associated with decrements in reading test scores, although this relationship was statistically significant only for NHB children.

The association between maternal age and reading test scores was nonlinear for both races (Fig. 1, *E* and *F*). Increasing maternal age appears detrimental to reading test scores until around 23 to 25 y, after which increasing maternal age was associated with improvements in reading test scores. This relationship appears more marked for NHW children.

**Fig. 2.**
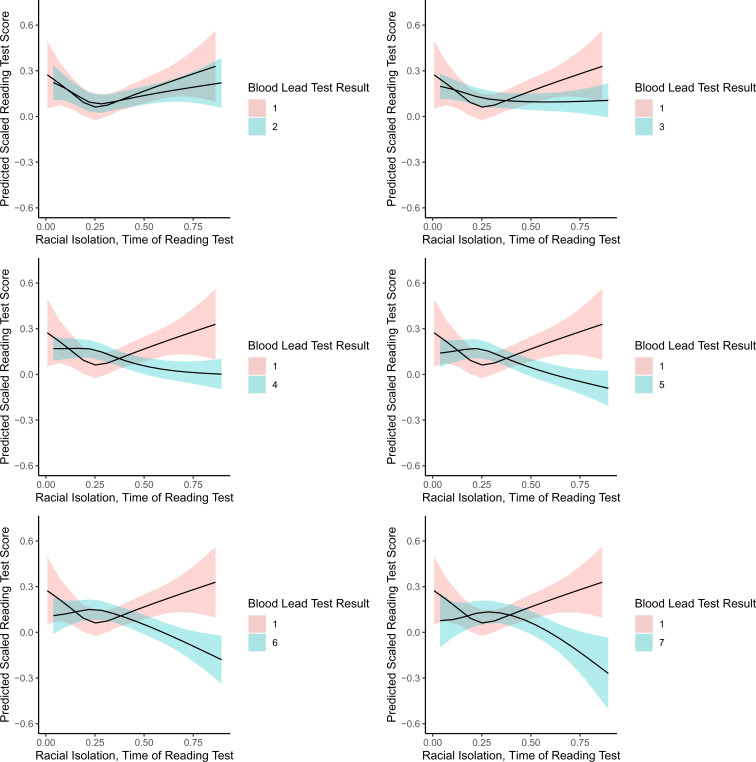
Interaction of BLL × RI_NHB_ at time of end-of-grade testing and reading test scores (NHB children).

Fig. 2 shows predicted scaled reading scores (*y*-axis) for NHB children across the range of RI_NHB_ values (*x*-axis) for BLLs ranging from 1 to ≥7 µg/dL Predicted test scores for NHB children with a BLL of 1 µg/dL are provided for comparison with predicted test scores of NHB children with higher BLLs. The highest BLL category, 7 µg/dL, includes individuals with BLLs of ≥7 µg/dL. Continuous covariates (e.g., birthweight percentile for gestational age, maternal age) are held at the mean, and categorical covariates (e.g., maternal educational attainment, maternal marital status, maternal smoking during pregnancy, child sex, computer use, and economic disadvantage) are set to their reference level. Fig. 2 shows that higher RI_NHB_ is only associated with lower reading test scores among NHB children with BLLs of at least 4 µg/dL The decrement in reading scores associated with higher levels of RI_NHB_ becomes even more marked at higher levels of lead exposure.

For linear variables, among NHB and NHW children, higher birthweight percentile for gestational age and higher maternal educational attainment were associated with higher reading test scores (Table 3). Male sex and economic disadvantage were associated with lower reading test scores. Smoking during pregnancy was associated with statistically significant decrements in reading test scores among NHW children, but not NHB children; however, NHB mothers were half as likely as NHW mothers to report smoking during pregnancy. Having an unmarried mother at time of birth was associated with lower reading test scores among NHB children and, to a lesser degree, NHW children. Residing in an urban census tract at time of end-of-grade testing was associated with statistically significant decrements in reading test scores among NHB children, but not NHW children.

**Table 3.** Results of the generalized additive model regression for end-of-grade test scores in reading: linear variables

Linear variable	NHB children		NHW children	
	Parametric coefficient (95% CI)*	*P* value	Parametric coefficient (95% CI)	*P* value
Child characteristics				
Birthweight percentile for gestational age	0.001 (0.0003, 0.0017)	0.004	0.001 (0.0003, 0.0014)	0.002
Male sex	−0.213 (−0.251, −0.175)	<0.001	−0.103 (−0.132, −0.0738)	<0.001
Computer use				
None	−0.064 (−0.105, −0.0231)	0.002	−0.105 (−0.137, −0.0729)	<0.001
Some	Reference		Reference	
Always	−0.336 (−0.402, −0.270)	<0.001	−0.286 (−0.354, −0.218)	<0.001
Economic disadvantage^†^	−0.262 (−0.318, −0.206)	<0.001	−0.283 (−0.319, −0.248)	<0.001
Year of end-of-grade standardized test				
2010	Reference		Reference	
2011	−0.163 (−0.203, −0.122)	<0.001	−0.089 (−0.120, −0.0575)	<0.001
Maternal characteristics				
Educational attainment				
No high school diploma	Reference		Reference	
High school diploma	0.216 (0.165, 0.268)	<0.001	0.267 (0.221, 0.312)	<0.001
College diploma	0.552 (0.462, 0.642)	<0.001	0.693 (0.633, 0.753)	<0.001
Smoked during pregnancy	−0.001 (−0.0627, 0.0609)	0.977	−0.030 (−0.0685, 0.0087)	0.130
Not married	−0.107 (−0.156, −0.0581)	<0.001	−0.036 (−0.0761, 0.0032)	0.071
Neighborhood characteristics				
Urbanicity of census tract at time of end-of-grade test	−0.085 (−0.130, −0.0406)	<0.001	0.007 (−0.0255, 0.0388)	0.684

^*^CIs reported were calculated as ±1.96 × SE.

^†^Economic disadvantage is indicated by participation in the free/reduced-price lunch program at time of end-of-grade testing.

Results for models of reading test scores that adjusted for RI_NHB_ at time of birth are presented in *SI Appendix*, Table S1 and Figs. S1 and S2. Results did not differ substantively from those reported here.

### Mathematics Scores.

As with models for reading test scores, due to correlation between RI_NHB_ at time of birth and RI_NHB_ at time of standardized testing, we fit separate models to adjust for each of these variables. Results from race-stratified generalized additive models for math test scores that adjust for RI_NHB_ at time of standardized testing are presented here; corresponding results for models that adjust for RI_NHB_ at time of birth are presented in *SI Appendix*.

Briefly, in race-stratified generalized additive models for mathematics test scores that adjust for RI_NHB_ at time of standardized testing, higher BLL was negatively associated with math test scores among NHB and NHW children. Neighborhood RI_NHB_ at time of standardized testing was negatively associated with math test scores for NHW children only (*SI Appendix*, Table S2). Maternal age was variably associated with math test scores for both NHB and NHW children (i.e., the relationship between maternal age and math test scores was nonlinear). There was no evidence of an interactive effect between BLL and neighborhood RI_NHB_ at time of end-of-grade test for either NHB or NHW children.

The relationship between each of the smooth terms and scaled mathematics test scores is shown in *SI Appendix*, Fig. S3. The plots of BLL (*SI Appendix*, Fig. S3 *A* and *B*) were linear for both races and indicate that higher BLLs are associated with lower test scores.

Among NHB children, the association between RI_NHB_ at time of testing and math test scores was not significant. The corresponding plot (*SI Appendix*, Fig. S3) shows that the association was negative until high levels of RI_NHB_ (e.g., ≥0.70), but the CIs were wide at high levels of RI_NHB_ (*SI Appendix*, Fig. S3*C*). Among NHW children, neighborhood RI_NHB_ at time of standardized testing was linearly associated with lower math test scores (*SI Appendix*, Fig. S3*D*), and again, the CIs were wide at high levels of RI_NHB._

Among NHB children, increasing maternal age was associated with lower math test scores until around age 25, after which math test scores plateau or improve with maternal age, although the CIs were wide (*SI Appendix*, Fig. S3*E*). The relationship between maternal age and math test scores among NHW children was similar to that observed for reading test scores (*SI Appendix*, Fig S3*F*).

Among NHB and NHW children, higher birthweight percentile for gestational age and higher maternal education were associated with higher math test scores (*SI Appendix*, Table S2). Economic disadvantage and having an unmarried mother were associated with lower math test scores. Smoking during pregnancy was associated with statistically significant decrements in math test scores among NHW children, but not NHB children. Male sex was associated with lower and higher math test scores among NHB and NHW children, respectively. Residing in an urban census tract at time of end-of-grade testing was associated with decrements in math test scores among NHB children, but not NHW children.

Results for models of math test scores that adjusted for RI_NHB_ at time of birth are presented in *SI Appendix*, Table S3 and Fig. S4. Results were generally similar to those presented here; that is, higher BLL was negatively associated with math test scores and maternal age was variably associated with math test scores for both NHB and NHW children. However, among NHB children, higher neighborhood RI_NHB_ at time of birth was associated with lower math test scores, while no association was observed between RI_NHB_ at time of birth and math test scores among NHW children. There was no evidence of an interactive effect between BLL and neighborhood RI_NHB_ at time of birth for either NHB or NHW children.

## Discussion

We constructed a longitudinally linked spatiotemporal dataset that tracks children from time of birth to time of fourth-grade end-of-grade standardized testing by linking multiple statewide administrative datasets in North Carolina. Using generalized additive models, we estimated nonlinear associations, and potential interactions, between neighborhood and environmental exposures and fourth-grade standardized test scores in reading and mathematics. A priori, we decided to include an interaction term between environmental (e.g., lead) and neighborhood (e.g., RI_NHB_) exposures to test the hypothesis that exposure to RI_NHB_ has potential to augment the adverse effects of lead exposure.

In this population-based sample, we observed that NHB children are more likely to experience economic disadvantage, have higher BLLs, reside in racially isolated neighborhoods, and have mothers who did not graduate from high school and are unmarried at time of birth; NHW children are more likely to have mothers who reported smoking during pregnancy. Thus, NHB children are more likely than NHW children to experience multiple adverse exposures. This underscores the importance of detecting and appropriately modeling interactions between multiple exposures, because these exposures have the potential to synergistically combine to adversely affect outcomes. Even without synergistic effects, the accumulation of adverse exposures that characterize this study population—and likely other groups of children—can be particularly detrimental to child cognitive and developmental health.

Critically, we did observe evidence of an interaction between BLL and RI_NHB_ on reading test scores among NHB children. NHB children with low BLLs (e.g., ≤3 µg/dL) who reside in high RI_NHB_ neighborhoods have similar reading test scores compared to their counterparts with low BLLs in low RI_NHB_ neighborhoods. In contrast, NHB children with high BLLs (e.g., ≥4 µg/dL) in high RI_NHB_ neighborhoods have significantly lower reading test scores compared to their counterparts with high BLLs in low RI_NHB_ neighborhoods.

This interaction between BLL and RI_NHB_ was observed for reading scores only among NHB children. However, the hypothesized mechanisms for this interaction (i.e., that depauperate environments and chronic stressors exacerbate the adverse effects of environmental exposures on health and development) should in theory apply to all children. However, NHB and NHW children are not equally exposed; if NHW children were exposed to high RI_NHB_ neighborhoods, an interaction between BLL and RI_NHB_ might be observed among NHW children as well. The vast majority of NHW children live in low(er) RI_NHB_ neighborhoods, so in this analysis, we may not have the exposure distribution and contrast necessary to detect an interaction. Moreover, even if NHW children were equally exposed to, for example, RI_NHB_, NHW children may be more likely than NHB children to benefit from cognition-, developmental-, or health-promoting exposures or access to resources that exert a protective effect. This warrants additional investigation.

Importantly, there is no “optimal” level of RI_NHB_, and we purposefully chose a nonlinear modeling approach to allow us to investigate how varying levels of RI_NHB_ might affect outcomes. The generalized additive modeling approach used here includes independent variables with nonparametric functions, subject to the constraint that the nonparametric effects additively combine. In this way, generalized additive models are an alternative to assuming global linearity by default. Had a more standard, linear modeling approach been used, the interaction between lead and RI_NHB_ might have been missed. In fact, in earlier work using a similarly structured dataset and linear models, we tested for but did not detect an interaction between lead exposure and RI_NHB_ (9).

This study has several limitations. While we observed associations between lead, RI, and educational outcomes, we cannot infer causality. The study sample used here, though population based, is not perfectly representative of North Carolina’s population. For example, we excluded children of mothers with a residential address at time of birth that could not be found in a reference dataset. Children removed from the analysis because their addresses could not be geocoded may differ from those included in the analysis with respect to characteristics that relate to exposure and outcomes. In addition, the children in our dataset are likely biased toward those who are at greatest risk of lead exposure given blood lead surveillance strategies and associated testing patterns employed in the absence of a universal lead screening program in North Carolina. These issues could affect the generalizability of our results. We used one lead test result for each child instead of repeated measures, which are not common in North Carolina’s blood lead surveillance data. However, other studies examining lead exposure and developmental outcomes have also utilized single measures of lead exposure (6, 29–31). Models did not adjust for insurance status, but information on mothers’ insurance type is available in the North Carolina detailed birth records (DBRs) beginning in 2011, and thus could be used in future work utilizing later birth cohorts. Finally, our findings may be biased due to unmeasured confounding, although we attempted to mitigate potential bias by controlling for maternal- and child-level covariates. Several of our findings merit further research, including why an association was observed between math test scores and RI_NHB_ at birth but not RI_NHB_ at time of standardized testing among NHB children, and why the reverse was observed among NHW children (i.e., RI_NHB_ at time of standardized testing was associated with math test scores among NHW children, but RI_NHB_ at time of birth was not). The absence an interaction of BLL and RI_NHB_ on math test scores should also be investigated in future work.

Despite limitations, this study has important strengths. We developed a spatiotemporal dataset that connects multiple administrative datasets. We were able to evaluate associations between multiple exposures, interactions between those exposures, and standardized test scores, for both NHB and NHW children, due to the substantial sample size. We also examine exposures occurring at various stages in the life course, which may be especially important given evidence suggesting that adverse effects of childhood lead exposure (8) and neighborhood conditions persist (12, 32), making the cumulative effect of these combined exposures of particular interest. For example, a study of verbal ability in African American children in Chicago, Illinois, concluded that the effects of neighborhood on verbal ability in children were “not instantaneous, but rather manifested several years later” (12). Our modeling approach offers the ease of interpretation associated with linear regression techniques but the flexibility of nonparametric methods (33). Finally, this work is also responsive to calls for prioritizing research that leverages population-level databases with information on location, childhood health, and developmental outcomes to better understand how neighborhoods shape health and development (11).

Increasingly, evidence suggests that cognitive, developmental, and health outcomes and disparities in these outcomes in adulthood relate to early life experiences and outcomes, including educational outcomes (34). Minh et al. (11) describe biological embedding as a process in which “social and environmental experiences in a child's early years are theorized to shape physiological changes that have lifelong protective or detrimental effects on children's learning, behavior, health and wellbeing” (35). Lead exposure and residence in racially segregated neighborhoods, which in turn affect educational outcomes, may shape later-life health and disease. We take this framework and show that the cumulation of exposures associated with living in certain neighborhoods combines to shape educational outcomes. We demonstrate that, among NHB children, there is an interaction between lead exposure and neighborhood RI on standardized test scores in reading, which would have been missed under a standard linear modeling approach; and that NHB children are more likely to have multiple adverse exposures. Thus, truly attacking the achievement gap will require interventions beyond what and how we teach in schools, including attention to the places and circumstances that characterize a child’s home environment.

## Materials and Methods

### Data.

The analysis dataset for this study was created by linking three administrative databases for the State of North Carolina: DBRs, blood lead surveillance data, and end-of-grade standardized testing data.

#### DBRs.

The DBRs were obtained from the Vital Statistics Department of the North Carolina State Center for Health Statistics. The DBRs include information on date of birth, location of birth, maternal characteristics (e.g., health, demographics, obstetrical history, residential address) and infant characteristics (e.g., gestational age, sex) for all documented live births in North Carolina. Validation studies have shown that birth certificate data has high accuracy, especially for variables describing demographic characteristics and birth outcomes (36, 37).

#### Blood lead surveillance data.

Blood lead surveillance records were obtained from the Childhood Lead Poisoning Prevention Program of the Children’s Environmental Health Unit, Department of Health and Human Services. Blood lead surveillance data include information on the child (e.g., name, age, test date, BLL, and residential address). The limit of detection for blood lead is 1 μg/dL and BLLs are recorded as integer values. Children with BLLs below the limit of detection were given a value of 1 μg/dL. Children should have been screened for lead exposure if their parents responded “yes” or “don’t know” to questions on the Centers for Disease Control and Prevention Lead Risk Assessment Questionnaire (38), or if they were Medicaid participants.

#### End-of-grade standardized testing data.

End-of-grade standardized test score data were obtained from the NC Education Research Data Center of Duke University. At the end of each academic year, North Carolina children enrolled in public schools and in grades 3 to 8 are administered standardized assessments of reading and mathematics. These are “curriculum-based multiple-choice achievement tests … specifically aligned to the *North Carolina Standard Course of Study*” (39). End-of-grade tests consist of multiple-choice questions that assess cognition, critical stance, interpretation, and connections (Reading) and numeration, numerical operations, geometry, patterns, relationships, functions, statistics, and probability (Mathematics) (39). These data also include information such as the child’s name and birth date as well as socioeconomic and demographic data, information on English proficiency, and school and school district identifier, among others.

Access to, management, and analysis of these data are governed by data use agreements and an Institutional Review Board–approved research protocol at the University of Notre Dame.

### Linking Datasets.

The linking of the DBR, blood lead surveillance data, and standardized test score datasets is described in detail elsewhere (9). Briefly, linking methods used different combinations of variables and match strength requirements to identify children across the three datasets, and they were developed to ensure accuracy while maximizing the number of records linked.

The initial DBR included 118,462 unique infants born to mothers in North Carolina between January 1, 2000 and December 31, 2000. Of these, 100,395 (84.7%) were street-geocoded, then linked to a 2000 census tract at time of birth. Mothers included in our analysis were between the ages of 15 and 44 y (excluded 331 records). We restricted to individuals who were singleton live births (excluded 3,164 records) without congenital anomalies (excluded 885 records). Children had a gestational age at delivery between 24 and 42 wk (excluded 343 records) and were born to self-reported NHW, NHB, and Hispanic mothers (excluded 3,464 records). Of the 92,208 births meeting the above criteria, 62,110 (67.4%) were successfully matched to a fourth-grade reading and math test score in 2010 to 2011 (encompassing two academic years: 2010/2011 and 2011/2012). Of the 62,110 births (67.4%) that were successfully matched to a fourth-grade reading and math test score in 2010 to 2011, 52,045 (83.8%) were geocoded at time of standardized testing. Of the 52,045 children with both geocoded birth and geocoded education records, 31,014 (59.6%) were linked with at least one lead test result. If a child had more than one education record (end-of-grade tests were administered more than once to some students), we retained all results in the analysis dataset but used the chronologically first test result in the analysis. We chose to use the chronologically first test result because, with few exceptions, this means that the child is being assessed at the same time as their peers. Retesting could occur for any number of reasons and a retested child may score higher after having additional time or interventions to master the material. If a child had more than one lead test result, we retained all results in the analysis dataset but used the maximum BLL in the analysis.

### Study Sample.

There were 31,014 children linked across DBR (in 2000), lead screening (2000 to 2011), and standardized testing datasets (2010 to 2011), and geocoded at time of birth and standardized testing. We restricted to children who were born to self-reported NHW and NHB mothers (excluded 3,863 records); did not have limited English proficiency, as it can be complicated to interpret test scores among young children for whom English is their second language (excluded 195 records); and had a BLL ≤80 μg/dL (excluded 3 records). We also removed 1,254 records (4.7%) with missing values for maternal education, tobacco use, child’s computer use at home, and years of end-of-grade tests variables. Our final analysis dataset included 25,699 children.

We compared characteristics of the DBR for all children born in 2000 (*n* = 100,327) with 1) children in the initial dataset of linked births, lead, and education records (*n* = 31,014) and 2) children in the final dataset of linked births, lead, and education records, i.e., the dataset after all exclusion criteria were applied (*n* = 25,699) in *SI Appendix*, Table S4. Reading and math scores were similar between the initial and final linked datasets. Birthweight percentile for gestational age was higher in the DBR (median = 47.7) compared to the initial and final linked datasets (median = 45.7). There were no Hispanic, non-Hispanic Asian/Pacific Islander, or non-Hispanic other children in the final dataset. NHB and NHW children were 23.8% and 62.7% of the DBR, respectively, and 38.6% and 61.4% of the final linked dataset, respectively. Hispanic children represented 9.9% of the DBR and 12.5% of the initial linked dataset. A higher proportion of mothers reported smoking during pregnancy in the final linked dataset (17.8%) compared to the initial linked dataset (16.1%) and the DBR (13.1%). Maternal age at time of child’s birth in the initial and final linked datasets was younger than in the DBR (25.0 vs. 27.0 y). Maternal educational attainment was lower in the final linked dataset compared to the DBR; for example, 21.3% of mothers had less than high school education in the final dataset compared to 20.4% in the DBR, and 17.4% of mothers had a college degree in the final dataset compared to 26.3% in the DBR. Proportions of mothers who were unmarried at time of birth were higher in the initial (43.3%) and final (44.1%) linked datasets compared to the DBR (31.2%).

#### Neighborhood RI.

We calculated RI index values at the census tract level based on 2000 and 2010 Census data using a previously derived local, spatial measure of RI (20), which is derived from the global spatial isolation index developed by Reardon and O’Sullivan (40). We calculated tract-level RI scores by accounting for the population composition in the index tract along with adjacent tracts. In calculating spatial indices, edge effects may occur when neighboring tracts located outside the study area are ignored, thus distorting the index values assigned to bordering tracts within the study area. We thus included neighboring tracts located in surrounding counties in our adjacency structure. RI_NHB_ ranges from 0 to 1: individuals living in a neighborhood environment that is nearly all non-NHB individuals will have a RI_NHB_ value that is close to 0. In contrast, individuals living in a neighborhood environment that is nearly all NHB individuals will have a RI_NHB_ value that is close to 1.

Each child in the final analysis dataset was assigned a RI_NHB_ value at time of birth and time of standardized testing. RI_NHB_ at birth was assigned based on the child’s tract of residence at time of birth (obtained from the DBR), using RI_NHB_ calculated from 2000 Census data. RI_NHB_ at time of standardized testing was assigned based on the child’s tract of residence at time of testing (obtained from the standardized testing data), using RI_NHB_ calculated from 2010 Census data.

#### Urbanicity.

Urbanicity was determined using primary and secondary rural urban commuting area (RUCA) codes, which delineate metropolitan, micropolitan, small town, and rural commuting areas (41). Developed by researchers at the US Department of Agriculture in collaboration with the Office of Rural Health Policy and the Rural Health Research Center, RUCA codes use measures of population density, urbanization, and size and direction of primary (largest) daily commuting flows to determine the degree of urbanicity of US census tracts. RUCA codes were used to classify tracts as either urban or rural (42).

### Statistical Analysis.

We used individual-level continuous reading and mathematics end-of-grade standardized fourth-grade test scores as dependent variables, modeled separately. Generalized additive models were used to estimate associations between environmental and social exposures on standardized test scores as well as interactions between environmental and social exposures. Generalized additive models are an alternative to assuming global linearity by default. This class of models is subject to the constraint that nonparametric effects additively combine, thus providing the straightforward interpretation of linear regression but the flexibility of nonparametric methods. Interaction effects are modeled as smooth functions of the continuous variable(s) and constrained to be orthogonal to the main effects, which keeps the interaction distinct from each main effect (43).

The environmental exposure of interest was lead exposure (measured by BLLs) and the neighborhood exposure of interest was (census tract-level) RI_NHB_ at time of birth and time of standardized testing. BLL and RI_NHB_ were treated as continuous variables.

In preliminary analyses, we observed that NHB and NHW children had different covariate distributions (Table 1). Thus, we chose to fit race-stratified models of standardized test scores, separately for reading and mathematics, that adjusted for maternal and child-level characteristics. Maternal characteristics obtained from the DBR included age (years), educational attainment (less than high school [i.e., <12th grade], completed high school [i.e., 12th grade], and completed college [i.e., ≥16 y of education]), marital status, and smoking during pregnancy. We adjusted for child characteristics as well, including male sex, birthweight percentile for gestational age (an indicator of fetal growth and newborn health) (44–46), computer use at home (none, some, daily/always), economic disadvantage (participation in the free/reduced-price lunch program) at time of end-of-grade testing, and urbanicity of census tract of residence at time of testing.

To investigate whether neighborhood racial segregation augments (or mitigates) adverse effects of lead exposure, we considered a model specification that included an interaction term between BLL and neighborhood RI_NHB_. Results from adjusted models with an interaction term (BLL × RI_NHB_) are presented if the interaction was significant (*P* < 0.05); otherwise, results from the adjusted model without an interaction term are presented.

All statistical analyses were performed using R version 3.5.0 (47). Models were fit with the Mixed GAM Computation Vehicle with Automatic Smoothness Estimation (mgcv) package (48).

## Supplementary Material

Supplementary File

Supplementary File

## Data Availability

The measure of RI is constructed from publicly available census data and is included with this manuscript as Dataset S1. Code files to replicate results reported are also included with this manuscript in *SI Appendix*, *Appendixes A*–*F*. Access to the DBRs, lead vital statistics data, and educational test score data described in this research is restricted and governed by data use agreements and protocols reviewed and approved by the Institutional Review Board at the University of Notre Dame.
