# Supplementary material for: Racial residential segregation shapes the relationship between early childhood lead exposure and fourth-grade standardized test scores
Source: Proc Natl Acad Sci U S A. 2022 Aug 15;119(34):e2117868119. doi: 10.1073/pnas.2117868119 (PMC9407651; doi:10.1073/pnas.2117868119)
Supplement: Supplementary File [file pnas.2117868119.sapp.pdf]

PNAS
www.pnas.org

Supplementary Information for

Racial residential segregation shapes the relationship between early childhood lead exposure and 4th grade standardized test scores

Mercedes A. Bravo,[1,2,*] Dominique Zephyr,[2] Daniel Kowal,[3] Katherine Ensor,[3] Marie Lynn Miranda[2,4]

[1] Global Health Institute, Duke University, Durham, NC, USA (mercedes.bravo@duke.edu)
[2] Children's Environmental Health Initiative, University of Notre Dame, South Bend, IN, USA. (pzephyr@nd.edu)
[3] Department of Statistics, Rice University, Houston, TX, USA. (daniel.kowal@rice.edu; ensor@rice.edu)
[4] Department of Applied and Computational Mathematics and Statistics, University of Notre Dame, South Bend, IN, USA. (mlm@nd.edu)

[*] Corresponding author: Mercedes A. Bravo, Global Health Institute, Duke University. 310 Trent Dr, Durham, NC 27708. 919-613-6163. mercedes.bravo@duke.edu

This PDF file includes:

Figures S1 to S4
Tables S1 to S4
Appendix A-F

Figure S1. Smoothed variables and predicted scaled reading test scores in the model adjusting for RI_{NHB} at time of birth

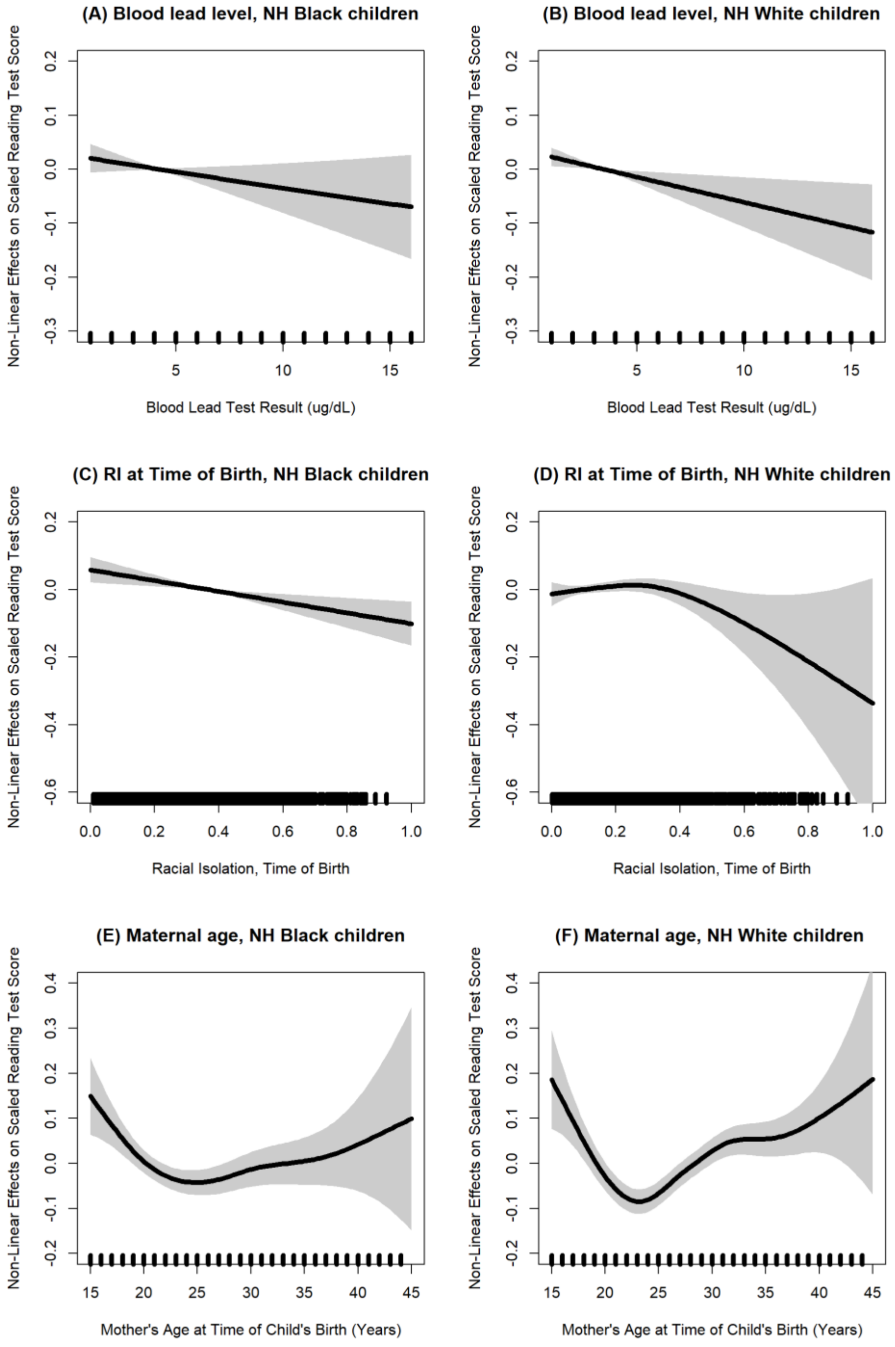

Figure S2. Interaction: blood lead level x RI_{NHB} at time of birth and reading test scores, NH Black children

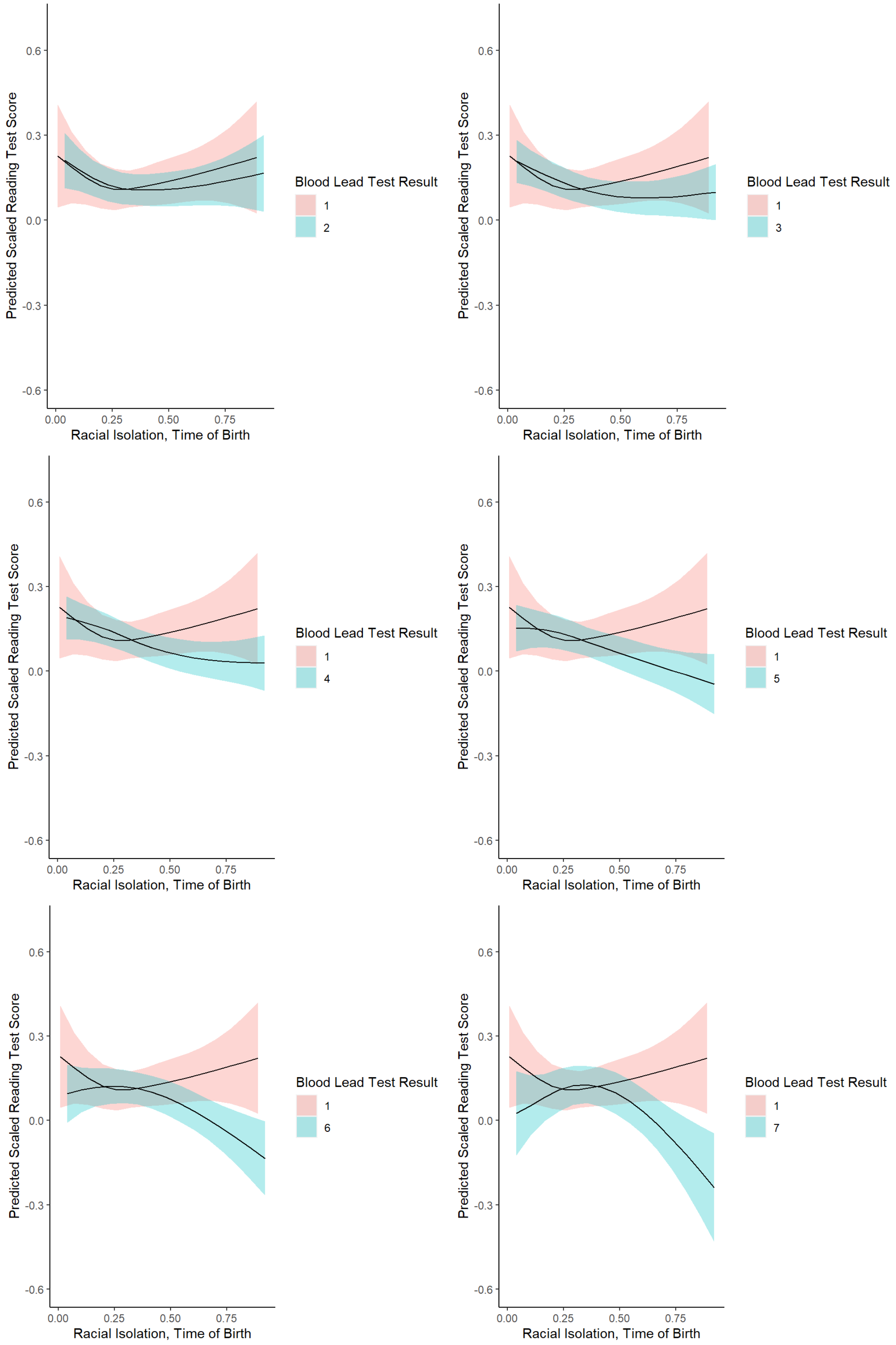

Figure S3. Smoothed variables and predicted scaled mathematics test scores in the model adjusting for RI_{NHB} at time of standardized test

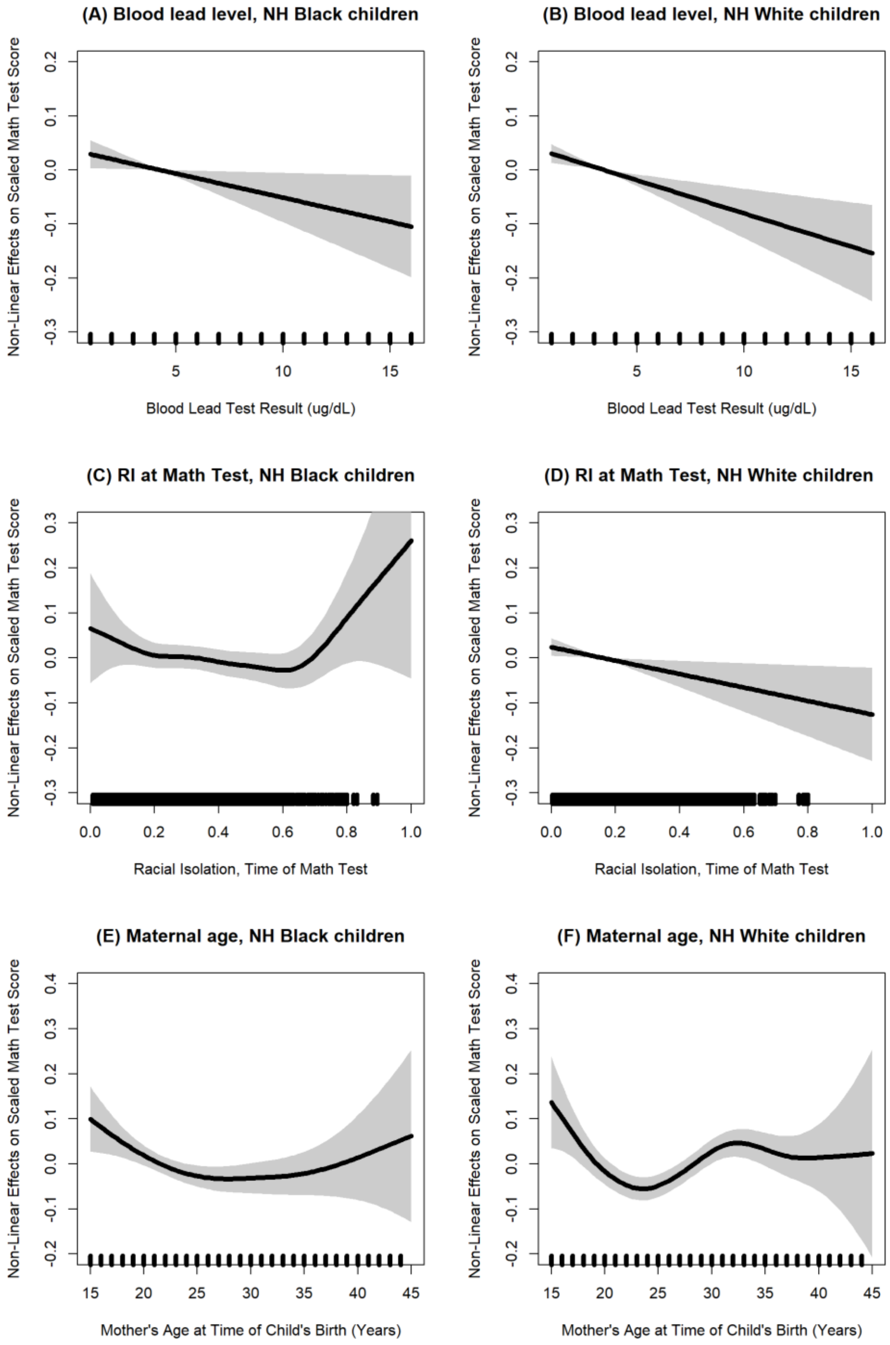

Figure S4. Smoothed variables and predicted scaled mathematics test scores in the model adjusting for RI_{NHB} at time of birth

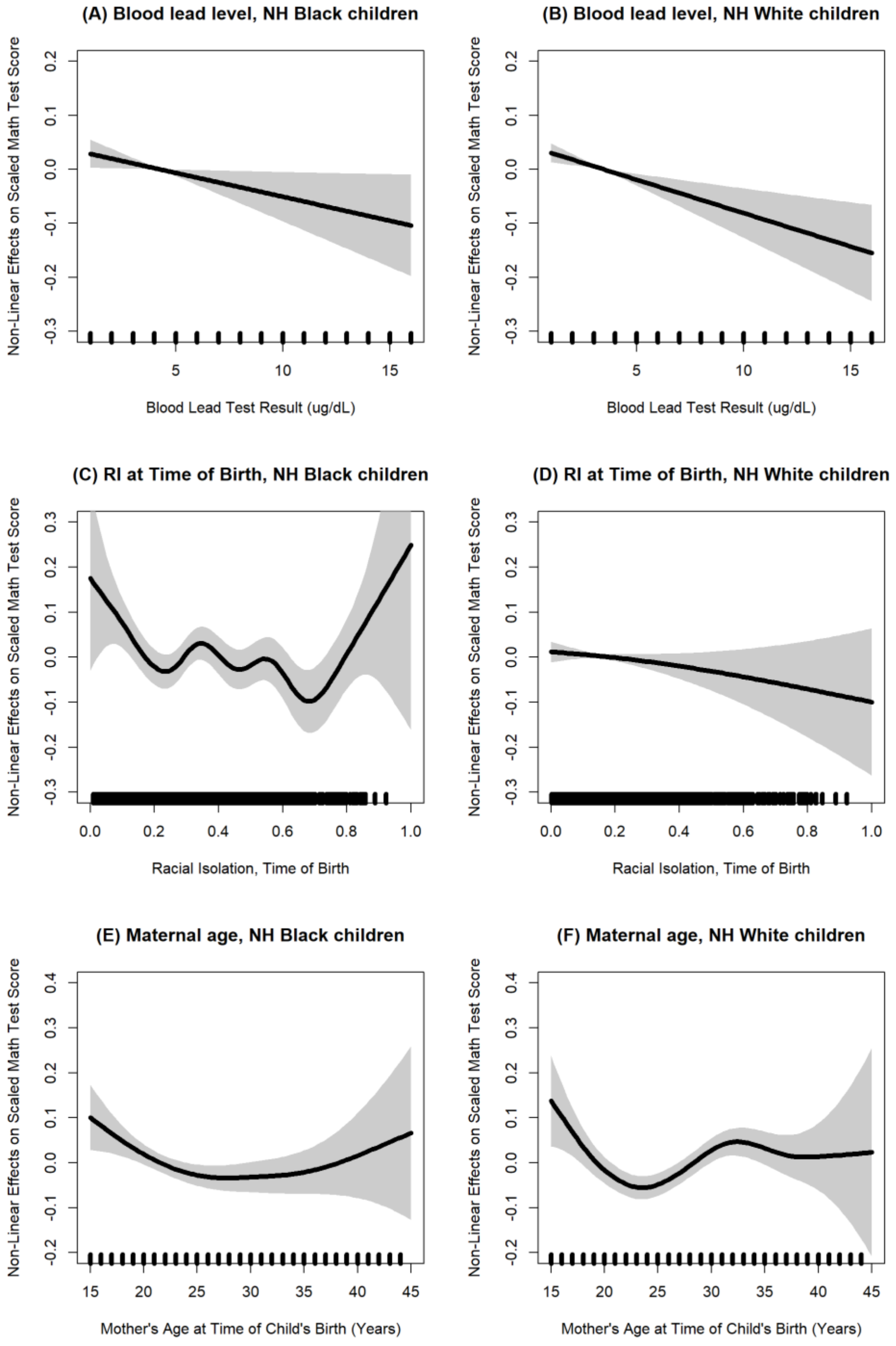

Table S1. Results of the Generalized Additive Model (GAM) regression for standardized test scores in reading (adjusting for RI_{NHB} at time of birth)

Linear variables[a,b]

	**NH Black children**		**NH White children**	
Variable	Parametric coefficient (95% confidence interval)	P-value	Parametric coefficient (95% confidence interval)	P-value
**Child characteristics**				
Birthweight percentile for gestational age	0.001 (0.0003, 0.0017)	0.003	0.001 (0.0003, 0.0014)	0.002
Male sex	-0.213 (-0.250, -0.175)	<0.001	-0.104 (-0.133, -0.0744)	<0.001
Computer Use				
None	-0.064 (-0.105, -0.0233)	0.002	-0.104 (-0.136, -0.0721)	<0.001
Some	Reference		Reference	
Always	-0.336 (-0.402, -0.2697)	<0.001	-0.286 (-0.354, -0.218)	<0.001
Economic disadvantage	-0.262 (-0.319, -0.2061)	<0.001	-0.282 (-0.317, -0.247)	<0.001
Year of end-of-grade standardized test				
2010	Reference		Reference	
2011	-0.164 (-0.205, -0.124)	<0.001	-0.089 (-0.120, -0.0581)	<0.001
**Maternal characteristics**				
Educational attainment				
No high school diploma	Reference		Reference	
High school diploma	0.215 (0.164, 0.267)	<0.001	0.267 (0.221, 0.3121)	<0.001
College diploma	0.553 (0.463, 0.643)	<0.001	0.693 (0.633, 0.7531)	<0.001
Smoked during pregnancy	0.001 (-0.061, 0.0626)	0.979	-0.030 (-0.0685, 0.0087)	0.129
Not married	-0.106 (-0.155, -0.0569)	<0.001	-0.037 (-0.0771, 0.0021)	0.063
**Neighborhood characteristics**				
Urbanicity of census tract at time of end-of-grade test	-0.088 (-0.1323, -0.0428)	<0.001	0.009 (-0.0233, 0.0413)	0.584

Nonlinear variables[c]

	**NH Black children**			**NH White children**		
	Effective degrees of freedom	P-value	F-statistic	Effective degrees of freedom	P-value	F-statistic
Blood lead level	1.042	0.135	2.273	1.006	0.008	6.974
RI at time of birth	1.003	0.002	9.709	2.536	0.111	1.958
Maternal age	3.585	0.003	3.875	5.289	<0.001	9.233
Blood lead level x RI_{NHB} at time of birth	5.025	0.010	2.751	–	–	–

[a] Confidence intervals reported were calculated as ±1.96*standard error. [b] Economic disadvantage is indicated by participation in the free/reduced price lunch program at time of end-of-grade testing. [c] Effective degrees of freedom (edf), F-statistic, and p-value are reported instead of coefficients for non-linear effects. Effective degrees of freedom represent the complexity of the smooth: an edf of 1 is equivalent to a straight line, an edf of 2 is equivalent to a quadratic curve, and so on, such that higher edf values describe more "wiggly" curves. Furthermore, an edf < k-1 indicates that k is sufficiently large. The F-statistic is a test statistic used in an analysis of variance to test overall significance, which produces the p-value. The table values are approximate, thus it is important to visualize your model to check the identified relationships (49).

Table S2. Results of the Generalized Additive Model (GAM) regression for standardized test scores in mathematics (adjusting for RI_{NHB} at time of standardized testing)

**Linear variables[a,b]**					
	**NH Black children**		**NH White children**		
Variable	Parametric coefficient (95% confidence interval)	P-value	Parametric coefficient (95% confidence interval)	P-value	
**Child characteristics**					
Birthweight percentile for gestational age	0.002 (0.0012, 0.0026)	<0.001	0.001 (0.0008, 0.0022)	<0.001	
Male sex	-0.072 (-0.110, -0.0337)	<0.001	0.076 (0.0379, 0.114)	<0.001	
Computer Use					
None	-0.068 (-0.109, -0.0266)	<0.001	-0.126 (-0.167, -0.0851)	<0.001	
Some	Reference		Reference		
Always	-0.321 (-0.388, -0.254)	<0.001	-0.238 (-0.304, -0.172)	<0.001	
Economic disadvantage	-0.241 (-0.297, -0.184)	<0.001	-0.264 (-0.320, -0.208)	<0.001	
Year of end-of-grade standardized test					
2010	Reference		Reference		
2011	-0.181 (-0.222, -0.140)	<0.001	-0.116 (-0.156, -0.0755)	<0.001	
**Maternal characteristics**					
Educational attainment					
No high school diploma	Reference		Reference		
High school diploma	0.197 (0.146, 0.249)	<0.001	0.245 (0.193, 0.297)	<0.001	
College diploma	0.540 (0.449, 0.6301)	<0.001	0.734 (0.644, 0.824)	<0.001	
Smoked during pregnancy	0.003 (-0.0591, 0.0654)	0.920	-0.039 (-0.101, -0.0224)	0.044	
Not married	-0.111 (-0.160, -0.0612)	<0.001	-0.057 (-0.107, -0.0082)	0.004	
**Neighborhood characteristics**					
Urbanicity of census tract at time of standardized test	-0.100 (-0.145, -0.0542)	<0.001	-0.013 (-0.0574, 0.0321)	0.438	
**Nonlinear variables[c]**					
	**NH Black children**			**NH White children**	

	Effective degrees of freedom	P-value	F-statistic	Effective degrees of freedom	P-value	F-statistic
Blood lead level	1.011	0.026	4.940	1.020	<0.001	12.060
RI at time of end-of-grade test	3.700	0.210	1.354	1.004	0.014	5.989
Maternal age	2.649	0.033	2.784	4.768	<0.001	4.962

[a] Confidence intervals reported were calculated as ±1.96*standard error. [b] Economic disadvantage is indicated by participation in the free/reduced price lunch program at time of end-of-grade testing. [c] Effective degrees of freedom (edf), F-statistic, and p-value are reported instead of coefficients for non-linear effects. Effective degrees of freedom represent the complexity of the smooth: an edf of 1 is equivalent to a straight line, an edf of 2 is equivalent to a quadratic curve, and so on, such that higher edf values describe more "wiggly" curves. Furthermore, an edf < k-1 indicates that k is sufficiently large. The F-statistic is a test statistic used in an analysis of variance to test overall significance, which produces the p-value. The table values are approximate, thus it is important to visualize your model to check the identified relationships (49).

Table S3. Results of the Generalized Additive Model (GAM) regression for standardized test scores in mathematics (adjusting for RI_{NHB} at time of birth)

Linear variables[a,b]

	NH Black children		NH White children	
Variable	Parametric coefficient (95% confidence interval)	P-value	Parametric coefficient (95% confidence interval)	P-value
**Child characteristics**				
Birthweight percentile for gestational age	0.002 (0.0012, 0.0026)	<0.001	0.001 (0.001, 0.002)	<0.001
Male sex	-0.071 (-0.109, -0.033)	<0.001	0.075 (0.0464, 0.104)	<0.001
Computer Use				
None	-0.069 (-0.110, -0.0279)	<0.001	-0.125 (-0.157, -0.093)	<0.001
Some	Reference		Reference	
Always	-0.321 (-0.388, -0.726)	<0.001	-0.238 (-0.306, -0.170)	<0.001
Economic disadvantage	-0.237 (-0.294, -0.180)	<0.001	-0.264 (-0.299, -0.229)	<0.001
Year of end-of-grade standardized test				
2010	Reference		Reference	
2011	-0.181 (-0.222, -0.1408)	<0.001	-0.116 (-0.147, -0.0852)	<0.001
**Maternal characteristics**				
Educational attainment				
No high school diploma	Reference		Reference	
High school diploma	0.194 (0.142, 0.246)	<0.001	0.245 (0.200, 0.291)	<0.001
College diploma	0.534 (0.444, 0.625)	<0.001	0.735 (0.675, 0.795)	<0.001
Smoked during pregnancy	0.003 (-0.0597, 0.0648)	0.936	-0.039 (-0.0776, -0.001)	0.044
Not married	-0.110 (-0.160, -0.0606)	<0.001	-0.059 (-0.0984, -0.0198)	0.003
**Neighborhood characteristics**				
Urbanicity of census tract at time of end-of-grade test	-0.098 (-0.143, -0.0522)	<0.001	-0.011 (-0.043, 0.0208)	0.493

Nonlinear variables[c]

	NH Black children			NH White children		
	Effective degrees of freedom	P-value	F-statistic	Effective degrees of freedom	P-value	F-statistic
Blood lead level	1.012	0.027	4.856	1.019	<0.001	12.247
RI at time of birth	6.846	0.007	2.478	1.254	0.247	1.055
Maternal age	2.680	0.030	2.831	4.778	<0.001	4.974

[a] Confidence intervals reported were calculated as ±1.96*standard error. [b] Economic disadvantage is indicated by participation in the free/reduced price lunch program at time of end-of-grade testing. [c] Effective degrees of freedom (edf), F-statistic, and p-value are reported instead of coefficients for non-linear effects. Effective degrees of freedom represent the complexity of the smooth: an edf of 1 is equivalent to a straight line, an edf of 2 is equivalent to a quadratic curve, and so on, such that higher edf values describe more "wiggly" curves. Furthermore, an edf < k-1 indicates that k is sufficiently large. The F-statistic is a test statistic used in an analysis of variance to test overall significance, which produces the p-value. The table values are approximate, thus it is important to visualize your model to check the identified relationships (49).

**Table S4. Comparison of characteristics of the initial North Carolina detailed birth records, the linked birth-lead-education dataset, and the final analysis dataset**			
	Initial NCDBR dataset (n=100,327) N (%)[a,b]	Initial linked dataset (N=31,014) N (%)[a]	Final linked analysis dataset (n=25,699) N (%)[a]
Reading test score, mean (SD)	--	346.13 (8.685)	346.81 (8.653
Math test score, mean (SD)	--	351.52 (8.342)	351.98 (8.370)
**Child characteristics**			
Birthweight percentile for gestational age, median (IQR)	47.70 (23.750-73.000)	45.70 (22.300-71.900)	45.70 (22.400-71.900)
Male sex	51,024 (50.9%)	15,652 (50.5%)	12,748 (49.6%)
Blood lead test result in ug/dL, median (IQR)	--	3.00 (2.000-5.000)	3.00 (2.000-5.000)
Residence in urban area at time of test	--	22,884 (73.8%)	18,733 (72.9%)
Computer use	-		
None	--	11,019 (37.0%)	9,100 (35.4%)
Some	--	16,648 (55.9%)	14,885 (57.9%)
Always	--	2,110 (7.1%)	1,714 (6.7%)
Economic disadvantage	--	19,951 (64.3%)	15,172 (59.0%)
Year of standardized test			
2010	--	19,702 (64.1%)	17,072 (66.4%)
2011	--	10,135 (33.0%)	8,627 (33.6%)
2012	--	882 (2.8%)	0 (0%)
**Maternal characteristics**[c]			
Race/ethnicity			
Hispanic	9,907 (9.9%)	3,863 (12.5%)	0 (0%)
NH Asian/Pacific Islander	2,514 (2.5%)	0 (0%)	0 (0%)
NH Black	23,883 (23.8%)	10,581 (34.1%)	9,909 (38.6%)
NH Other	1,070 (1.1%)	0 (0%)	0 (0%)
NH White	62,953 (62.7%)	16,570 (53.4%)	15,790 (61.4%)
Reported smoking during pregnancy	13,129 (13.1%)	4,981 (16.1%)	4,580 (17.8%)
Age at time of child's birth, median (IQR)	27.0 (22.0-31.0)	25.0 (21.0-30.0)	25.0 (21.0-30.0)
Educational attainment			
Less than high school	20,393 (20.4%)	8,746 (28.3%)	5,474 (21.3%)
High school diploma	53,327 (53.3%)	17,555 (56.7%)	15,756 (61.3%)
College diploma or higher	26,368 (26.3%)	4,639 (15.0%)	4,469 (17.4%)
Unmarried at time of birth	31,336 (31.2%)	13,670 (44.1%)	11,131 (43.3%)
**Neighborhood characteristics**			
Racial isolation at birth, median (IQR)	0.19 (0.094-0.31)	0.21 (0.11- 0.35)	0.21 (0.11- 0.34)
Racial isolation at standardized test, median (IQR)	--	0.21 (0.10- 0.35)	0.20 (0.093- 0.35)
[a]The cell count and percent are presented except in the case of blood lead test results, birth outcomes, and neighborhood characteristics, where the median (standard deviation) is provided, as indicated next to the variable name. [b] Dashes are provided for variables that were obtained from the lead surveillance data or standardized testing data, or were obtained based on residence at time of standardized testing (i.e., these variables are not available in the NC DBR). [c]Maternal variables are based on reported maternal characteristics at time of the child's birth.			
